# Nitric Oxide-Mediated Antioxidative Mechanism in Yeast through the Activation of the Transcription Factor Mac1

**DOI:** 10.1371/journal.pone.0113788

**Published:** 2014-11-25

**Authors:** Ryo Nasuno, Miho Aitoku, Yuki Manago, Akira Nishimura, Yu Sasano, Hiroshi Takagi

**Affiliations:** Graduate School of Biological Sciences, Nara Institute of Science and Technology, Ikoma, Nara, Japan; Temple University, United States of America

## Abstract

The budding yeast *Saccharomyces cerevisiae* possesses various defense mechanisms against environmental stresses that generate reactive oxygen species, leading to growth inhibition or cell death. Our recent study showed a novel antioxidative mechanism mediated by nitric oxide (NO) in yeast cells, but the mechanism underlying the oxidative stress tolerance remained unclear. We report here one of the downstream pathways of NO involved in stress-tolerance mechanism in yeast. Our microarray and real-time quantitative PCR analyses revealed that exogenous NO treatment induced the expression of genes responsible for copper metabolism under the control of the transcription factor Mac1, including the *CTR1* gene encoding high-affinity copper transporter. Our ChIP analysis also demonstrated that exogenous NO enhances the binding of Mac1 to the promoter region of target genes. Interestingly, we found that NO produced under high-temperature stress conditions increased the transcription level of the *CTR1* gene. Furthermore, NO produced during exposure to high temperature also increased intracellular copper content, the activity of Cu,Zn-superoxide dismutase Sod1, and cell viability after exposure to high-temperature in a manner dependent on Mac1. NO did not affect the expression of the *MAC1* gene, indicating that NO activates Mac1 through its post-translational modification. Based on the results shown here, we propose a novel NO-mediated antioxidative mechanism that Mac1 activated by NO induces the *CTR1* gene, leading to an increase in cellular copper level, and then Cu(I) activates Sod1. This is the first report to unveil the mechanism of NO-dependent antioxidative system in yeast.

## Introduction

The budding yeast *Saccharomyces cerevisiae* is an important microorganism not only as a model of higher eukaryotes but also in the fermentation industry. *S. cerevisiae* cells possess numerous defense mechanisms against various stresses [1]. During fermentation processes, environmental stresses, such as high temperature, high concentrations of ethanol, high osmotic pressure, desiccation, and freezing, induce the generation of reactive oxygen species (ROS) in the cell. The generated ROS causes severe damage to the intracellular molecules, including nucleic acids, proteins, and lipids, leading to cell death [2]–[7].

We recently revealed a novel antioxidative mechanism mediated by nitric oxide (NO) in yeast, which lacks the mammalian NO synthase (NOS) orthologue in the genome [8]. Our results indicated that NO produced in response to high-temperature stress that induces intracellular ROS generation conferred stress tolerance to yeast cells. Interestingly, we found that the yeast essential protein Tah18, which is a putative oxidoreductase, is involved in NO production under high-temperature stress conditions in an arginine-dependent manner. However, the mechanism underlying the oxidative stress tolerance by NO remained unclear.

NO is a diffusible free radical and a ubiquitous signaling molecule involved in the regulation of many cellular functions in animals, plants and microorganisms [9]–[12]. In mammalian cells, NO activates soluble guanylate cyclase (sGC) and increases cGMP level. The produced cGMP acts as a second messenger regulating numerous cellular events, including vascular function and neurotransmission [13]. In addition, NO directly participates in the post-translational activation of proteins via *S*-nitrosylation [14]–[16]. It has been reported that *S*-nitrosylation plays important roles in regulating apoptosis [17] and cardiac muscle function [14]. In *Escherichia coli*, *S*-nitrosylation of the transcription factor OxyR, which regulates many antioxidative genes, activates its ability for transcriptional activation [18]. Furthermore, NO is required to protect plant cells against iron-mediated oxidative stress [19]. In yeast, NO may be involved in various stress-response systems, including H_2_O_2_-induced apoptosis [20] and copper metabolism [21].

In *S. cerevisiae*, copper uptake is mediated by many steps: first, the cupric reductases encoded by the *FRE1*, *FRE2*, and *FRE7* genes reduce Cu(II) to Cu(I) at the surface of cell, and then Cu(I) is transported by the plasma membrane high affinity transporters (Ctr1, Ctr3) [22]–[26]. The imported Cu(I) is chelated by chaperones and transported to various cellular compartments [26]. Under copper starvation conditions, the genes for copper utilization are highly induced for the uptake of Cu(I) [27]. Such upregulation is dependent on the transcription factor Mac1, which is activated by releasing copper ions from its cysteine residues in response to copper starvation [28], [29]. It was also reported that the activation of Mac1 requires its phosphorylation [30]. On the other hand, yeast has a tolerance mechanism against copper toxicity mediated by the transcription factor Ace1, which is regulated in a manner similar to Mac1. Ace1 is activated by the binding of copper ions to its cysteine residues [31]. Copper homeostasis is important for tolerance to oxidative stress as well as freeze-thaw stress [32], whereas copper ion is a cofactor for the Cu,Zn-superoxide dismutase Sod1, which is one of the crucial antioxidative enzymes [7].

In this study, we report one of the downstream pathways of NO in yeast. We found that exogenous NO treatment increased the expression level of genes responsible for copper metabolism. Furthermore, our results indicate that Mac1 activation by NO under high-temperature stress conditions conferred stress tolerance to yeast cells and that Cu (I) ions incorporated through Ctr1 increased Sod1 activity. Thus, we propose a novel NO-mediated antioxidative mechanism though the activation of Mac1.

## Materials and Methods

### Strains, plasmids, and culture media

The yeast *S. cerevisiae* strains with a S288C background used in this study were wild-type BY4741 (*MATa his3Δ1 leu2Δ0 met15Δ0 ura3Δ0*), BY4741*Δmac1* (*MATa his3Δ1 leu2Δ0 met15Δ0 ura3Δ0 mac1::kanMx4*), BY4741*Δsod1* (*MATa his3Δ1 leu2Δ0 met15Δ0 ura3Δ0 sod1::kanMx4*), and BY4741*Δsod2* (*MATa his3Δ1 leu2Δ0 met15Δ0 ura3Δ0 sod2::kanMx4*) (Open Biosystems). The yeast *S. cerevisiae* strains with a Σ1278b background used in this study were wild-type L5685 (*MATa ura3-52 trp1*) and L5685*Δmac1* (*MATa ura3-52 trp1 mac1::kanMx4*). Strain BY4741MAC1-TAP, which expresses Mac1 fused with tandem affinity purification (TAP) tag containing calmodulin-binding protein and protein A at its C-terminus from the genome under the control of the original promoter, was used for ChIP. To complement auxotrophy of yeast strains, plasmids pRS414 (Stratagene) harboring *TRP1*, pRS415 (Stratagene) harboring *LEU2*, pRS416 (Stratagene) harboring *URA3*, and pRS416CgHIS3MET15 harboring *URA3*, *CgHIS3*, and *MET15* were used. The media used for the growth of *S. cerevisiae* were a synthetic minimal medium SD [2% (wt/vol) glucose, 0.17% (wt/vol) Bacto yeast nitrogen base without amino acids and ammonium sulfate (Difco Laboratories), and 0.5% (wt/vol) ammonium sulfate], a low-copper complete medium LCM [2% (wt/vol) glucose, 0.17% (wt/vol) Bacto yeast nitrogen base without copper, amino acids, and ammonium sulfate (Difco Laboratories), and 0.5% (wt/vol) ammonium sulfate], and a nutrient medium YPD [2% (wt/vol) glucose, 1% (wt/vol) yeast extract, and 2% (wt/vol) peptone].

### DNA microarray

*S. cerevisiae* BY4741 was cultivated in SD medium to the exponential growth phase (OD_600_ = 1.0), then a non-enzymatic NO donor, *S*-nitroso-*N*-acetylpenicillamine (SNAP), was added to the final concentration of 100 µM. As a control, dimethyl sulfoxide (DMSO) as the drug vehicle was also added. Yeast cells were collected at 0, 10, 30, and 60 min after addition of SNAP or DMSO. After washing the cells with 50 mM potassium phosphate buffer (pH 7.4), total RNA of each sample was extracted by RNeasy Mini Kit (Qiagen). The procedure for the extraction of total RNA was according to the manufacturer's instruction. The quality of the extracted RNA was confirmed with Bioanalyzer 2100 (Agilent Technologies). GeneChip(R) arrays (Affymetrix) were used as the DNA microarrays. DNA microarray analysis was performed with Bio Matrix Research. Statistical analysis after data acquisition and normalization of expression data was performed using GeneSpring (Agilent Technologies). For the pathway- or function-based category classification, the Munich Information Center for Protein Sequence (MIPS) was used. The dataset for microarray analysis has been submitted to Gene Expression Omnibus (GEO) (http://www.ncbi.nlm.nih.gov/geo/). The accession number is GSE61836.

### RT qPCR analysis

The primers used for the mRNA quantification are listed in Table S1. To examine the effect of SNAP treatment on gene expression, *S. cerivisiae* BY4741 and BY4741*Δmac1* cells were grown in SD medium at 25°C to OD_600_ of 1.0 and then subjected to SNAP treatment followed by the process for total RNA extraction. The cDNA synthesis and RT qPCR analysis was performed using High Capacity cDNA Reverse Transcription Kit (Applied Biosystems) and SYBR Green PCR Master Mix (Applied Biosystems), according to the manufacturer's instruction. The mRNA level of each gene was normalized to that of *ACT1* in the same sample. The cycle thresholds for each gene were normalized to *ACT1* and the relative induction fold compared to untreated cells was shown, for which 1.0 indicates no change in abundance. The values are the means of three independent experiments. To elucidate the effect of high-temperature stress on gene expression, *S. cerevisiae* L5685 and L5685*Δmac1* cells were grown in SD medium at 25°C to OD_600_ of 1.0 and then subjected to further incubation at 25°C or 39°C followed by the process for RT qPCR analysis described above. The mRNA level of each gene was normalized to that of *ALG9* in the same sample.

### ChIP analysis

*S. cerevisiae* BY4741MAC-TAP grown in SD medium or LCM medium were cross-linked by formaldehyde at 30°C for 15 min and then the reaction was stopped by addition of 125 mM glycine. Yeast cells were harvested, washed with and suspended in 50 mM HEPES-KOH (pH 7.5) containing 140 mM NaCl, 1 mM EDTA, 1% (wt/vol) Triton X-100, 0.1% sodium deoxycholate, Protease Inhibitor Cocktail (Sigma Aldrich), and 1 mM phenylmethylsulfonyl fluoride (PMSF) (IP buffer) and then disrupted with glass beads (0.5 mm diameter) in a Multi-Beads Shocker (Yasui Kikai). Immunoprecipitation was performed using IgG-sepharose beads (GE healthcare) equilibrated by IP buffer. After contaminant proteins were washed away with IP buffer, IP buffer with 500 mM NaCl, 50 mM Tris-HCl (pH 8.0) containing 250 mM LiCl, 0.5% NP-40, 0.5% sodium deoxycholate, and 1 mM EDTA (IP wash buffer) and 50 mM Tris-HCl (pH 8.0) containing 10 mM EDTA (TE), Mac1-TAP was eluted by the incubation in TE with 1% sodium dodecyl sulfate (SDS) at 65°C for 15 min and then further incubated at 68°C for 6 h for de-cross-linking. After deproteination with Proteinase K treatment followed by phenol-chloroform extraction and ethanol precipitation, the resultant DNA was used as a template for following PCR reaction using *EX Taq* DNA polymerase (Takara Bio). The primers used for the amplification of the promoter region were listed in Table S1. Densitometry using ImageJ [33] was performed for quantification of DNA binding to Mac1.

### Measurement of cell viability

*S. cerevisiae* L5685 or L5685*Δmac1* cells grown at 25°C in SD medium to OD_600_ of 1.0 were further incubated at 25°C or 39°C in the presence or absence of a mammalian NOS inhibitor, *N* ^G^-nitro-l-arginine methyl ester (NAME), and then cells were spread on YPD plates followed by further incubation at 30°C. The cell viability was expressed as percentages, calculated as follows: (the number of colonies after exposure to high temperature stress (39°C))/(the number of colonies after exposure to non-stress condition (25°C))×100.

### Determination of intracellular Cu(I) content

*S. cerevisiae* L5685 or L5685*Δmac1* cells grown at 25°C in SD medium to OD_600_ of 1.0 were further incubated at 25°C or 39°C in the presence or absence of NAME. After incubation, cells were collected, washed with and suspended in 50 mM potassium phosphate buffer (pH 7.4), then subjected to heat-shock treatment at 100°C for 15 min. After cooling on ice, 300 µl of supernatant was mixed with 300 µl of 500 mM ascorbic acid and 600 µl of 2 mM bathocuproine (BCS) and then incubated at 25°C for 10 min. The absorbance at 485 nm of the supernatant after centrifugation was measured and the intracellular total copper content was calculated as Cu (I) concentration using the extinction coefficient (ε = 1.2×10^4^ (M^−1^·cm^−1^)).

### Measurement of SODs activity

Total superoxide dismutases (SODs) activity was measured with SOD Assay Kit-WST (Dojindo) by kinetic assay following manufacturer's instruction. To examine the effect of high-temperature stress on SOD activity, *S. cerevisiae* L5685 or L5685*Δmac1* cells grown in SD medium at 25°C to OD_600_ = 1.0 were further incubated at 25°C or 39°C in the presence or absence of NAME. Then, cells were disrupted with glass beads (0.5 mm diameter) in a Multi-Beads Shocker. In order to distinguish the activity of Cu,Zn-SOD (Sod1) from Mn-SOD (Sod2), SODs activity was measured in the presence of 2 mM KCN, which inhibits the activity of Sod1 completely, therefore the residual activity indicates Sod2 activity. Then, the activity of Sod1 was calculated using total and Sod2 activity.

### Fluorescent microscopy for NO detection

*S. cerevisiae* L5685 or L5685*Δmac1* cells were grown in SD medium at 25°C to OD_600_ of 1.0, then 15 µM diaminofluorescein-FM diacetate (DAF-FM DA), which is a NO specific fluorescence probe, was added and incubated at 25°C for 1 h. After further incubation at 25°C or 39°C, cells were collected, washed with and suspended in 50 mM potassium phosphate buffer (pH 7.4). The fluorescence from the complex of DAF-FM DA with NO was observed by fluorescence microscopy Axiovert 200M microscope (Carl Zeiss) at the excitation wavelength of 495 nm and the fluorescence wavelength of 515 nm.

## Results

### DNA microarray analysis of the exogenous NO treatment

In our previous study, we proposed a novel antioxidative mechanism mediated by NO in yeast [8]. To obtain insights into the mechanism by which NO confers oxidative stress tolerance to yeast cells, we performed DNA microarray analysis of cells treated with NO. *S*-Nitroso-*N*-acetylpenicillamine (SNAP), which is a non-enzymatic NO donor, evokes *S*-transnitrosylation to other compounds such as amino acids or cysteine residues in a protein. Genes whose expression was significantly increased after exposure to SNAP for 10 min were extracted and classified by cellular functions using the MIPS database (http://mips.helmholtz-muenchen.de/genre/proj/yeast/). By this classification, we found that the category “heavy metal ion transport” had significantly high P values (1.22E-05). This category contains the genes involved in copper ion transport (*CTR1*, *FRE1*, and *FRE7*). Interestingly, it is known that the transcription of these genes is under the control of the transcription factor Mac1 [28], [34]. Takahashi *et al.* previously reported that copper ion homeostasis regulated by Mac1 is important for tolerance to freeze-thaw stress, which is one of the oxidative stresses [32]. Based on these findings, we speculated that there is some relevance between Mac1 and NO-mediated oxidative stress tolerance.

### Exogenous NO induces the transcription of the genes involved in copper metabolism under the control of the transcription activator Mac1

**Figure 1:**
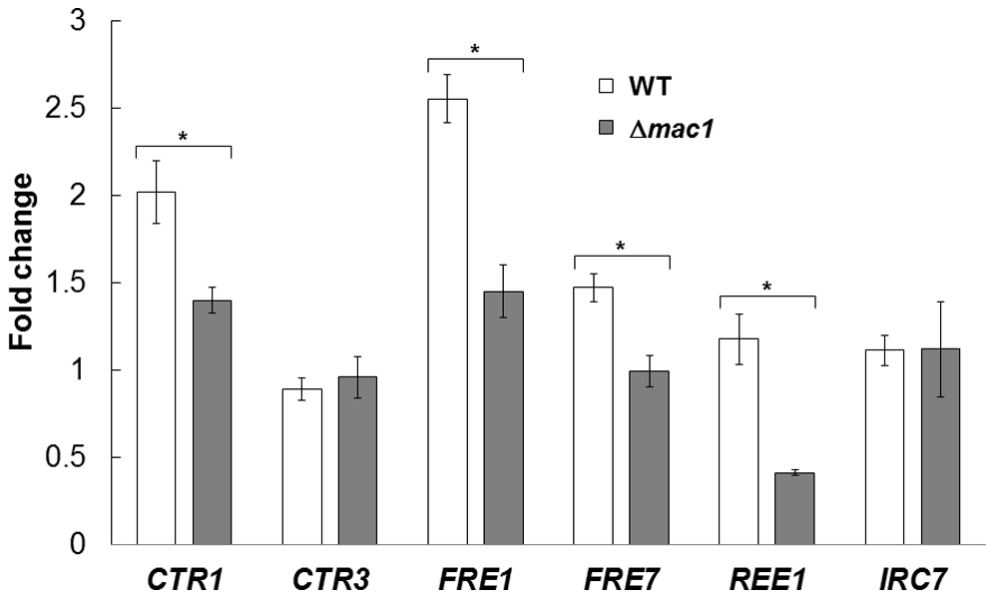
RT qPCR analysis of yeast cells treated with the exogenous NO. *S. cerevisiae* BY4741 or BY4741*Δmac1* cells treated or untreated with SNAP were subjected to RT qPCR analysis. The mRNA level of each gene was normalized to that of *ACT1* and the relative induction fold compared to untreated cells was shown. The values are the means and standard deviations of three independent experiments. *p<0.05 by Student's *t* test.

To confirm that exogenous NO activates the transcription of Mac1-regulated genes, we performed RT qPCR analysis of wild-type and mac1-disrupted cells treated with SNAP (Fig. 1). Among six Mac1-regulated genes examined [34], the *CTR1*, *FRE1*, and *FRE7* genes were significantly induced in wild-type cells in response to NO generated by SNAP treatment, although these increases were abolished when *MAC1* was disrupted. These results suggest that NO induces the Mac1-dependent activation of copper uptake and utilization in yeast cells.

### Exogenous NO facilitates the binding of Mac1 to the promoter region of target genes

**Figure 2:**
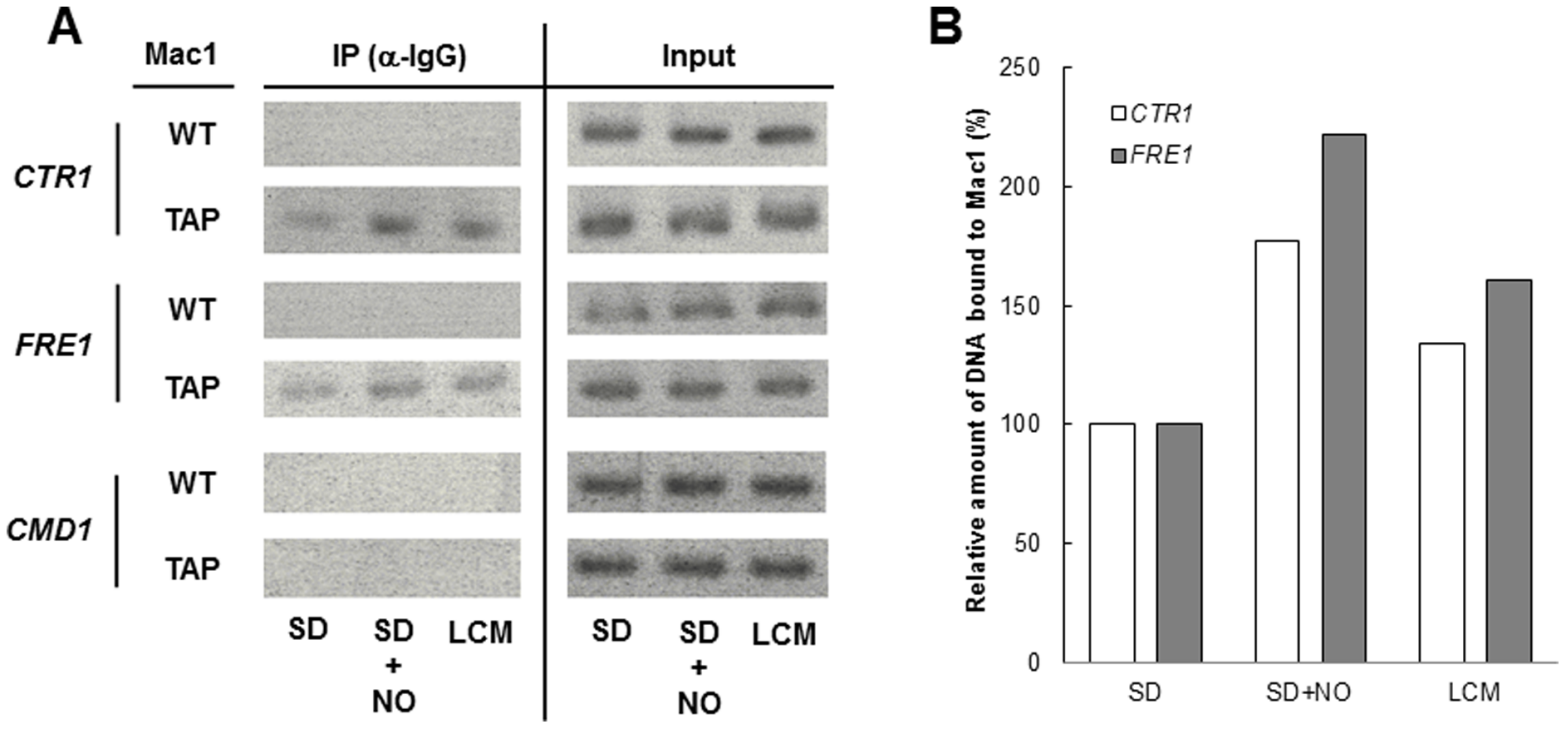
ChIP analysis of yeast cells treated with the exogenous NO. *S. cerivisiae* BY4741 and BY4741MAC1-TAP cells grown in SD medium with/without NO treatment or in LCM medium, which is the same as SD medium without supplementation of copper, were subjected to ChIP analysis. (A) Electrophoresis of amplified DNA corresponding to the promoter region of each gene. The *CMD1* gene, which is not under the control of Mac1, was used as a negative control. (B) Quantification of DNA binding to Mac1 by densitometry. The signal from immunoprecipitated DNA was divided by the signal from the corresponding input DNA. The resultant values were normalized as the value in SD medium is 100%.

It was previously reported that, under copper depletion conditions, the binding of Mac1 to upstream regulatory sequences of target genes is increased and the transcription of the Mac1-targeted genes is upregulated [29], [34]. To examine whether NO facilitates the binding of Mac1 to the target promoters, a chromatin immunoprecipitation (ChIP) assay was performed (Fig. 2A, B). In accordance with the previous report [29], the Mac1 binding to the *CTR1* and *FRE1* promoter was increased under copper depletion conditions in LCM medium. Similarly, when yeast cells were exposed to NO by SNAP treatment, an approximately two-fold increase in the binding of Mac1 to the *CTR1* and *FRE1* promoters was shown, while no binding occurred in the *CMD1* promoter, which is not regulated by Mac1. These results suggest that NO-activated Mac1 acquires the ability to bind the target promoter *in vivo*.

### NO produced under high-temperature stress conditions activates Mac1

**Figure 3:**
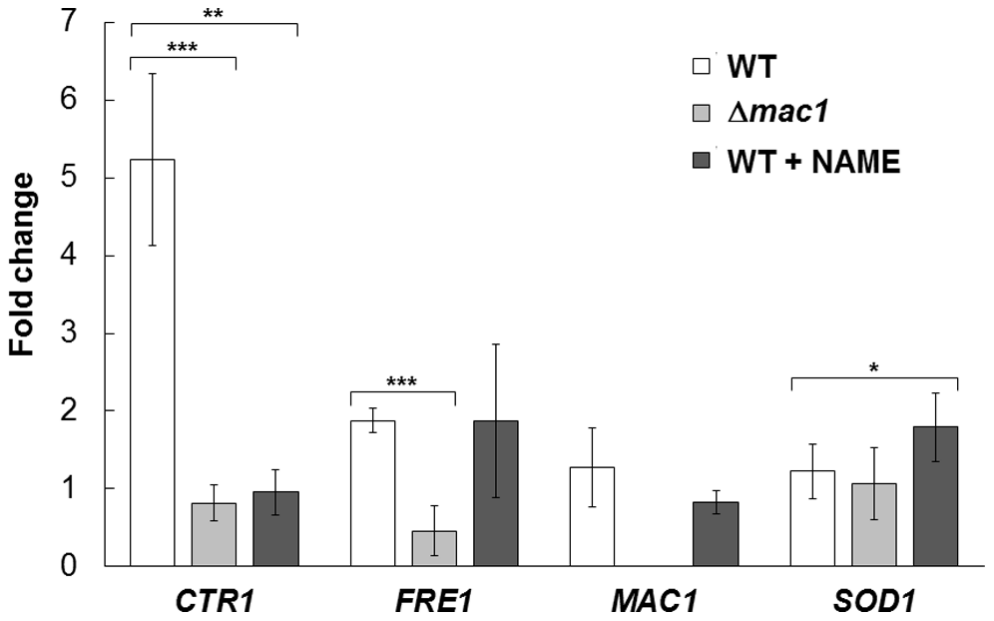
RT qPCR under high-temperature stress conditions. *S. cerevisiae* L5685 or L5685*Δmac1* cells were incubated at 39°C in the presence (100 mM) or absence of NAME followed by the process for RT qPCR analysis. The mRNA level of each gene was normalized to that of *ALG9* and the relative induction fold compared to cells incubated at 25°C was shown. The values are the means and standard deviations of four independent experiments. ***p<0.001; **p<0.01; *p<0.05 by Student's *t* test.

Our results shown above indicate that Mac1 is activated by NO and induces its target genes. We recently found that NO confers high-temperature stress tolerance to yeast cells [8]. Therefore, we hypothesized that Mac1 is involved in NO-mediated high-temperature stress-tolerance mechanism. Real-time quantitative PCR (RT qPCR) analysis was performed to examine whether Mac1 is also activated under high-temperature stress conditions (Fig. 3). The expression of *CTR1* was remarkably induced after exposure to high temperature (39°C) for 4 h, although this induction was abolished in *mac1*-dusrupted cells. Next, the effect of NAME on the induction of *CTR1* was examined. Temperature up-shift to 39°C induced NO production regardless of the presence or absence of *MAC1*, indicating that Mac1 functions in the downstream of NO production in yeast (Fig. S1). On the other hand, NAME treatment inhibited NO production under high-temperature stress conditions in both wild-type and *mac1*-disrupted cells (Fig. S1). Interestingly, the induction of *CTR1* in response to high temperature was canceled by NAME treatment. Considering that the exogenous NO induces *CTR1* in a Mac1-dependent manner (Fig. 1), these results indicate that stress-dependent induction of *CTR1* occurs through the activation of Mac1 by NO. Based on the fact that high-temperature stress did not increase the expression level of *MAC1* (Fig. 3), it was suggested that NO activates Mac1 through post-translational modifications, such as *S*-nitrosylation and phosphorylation.

### NO confers high-temperature stress tolerance to yeast cells in a Mac1-dependent manner

**Figure 4:**
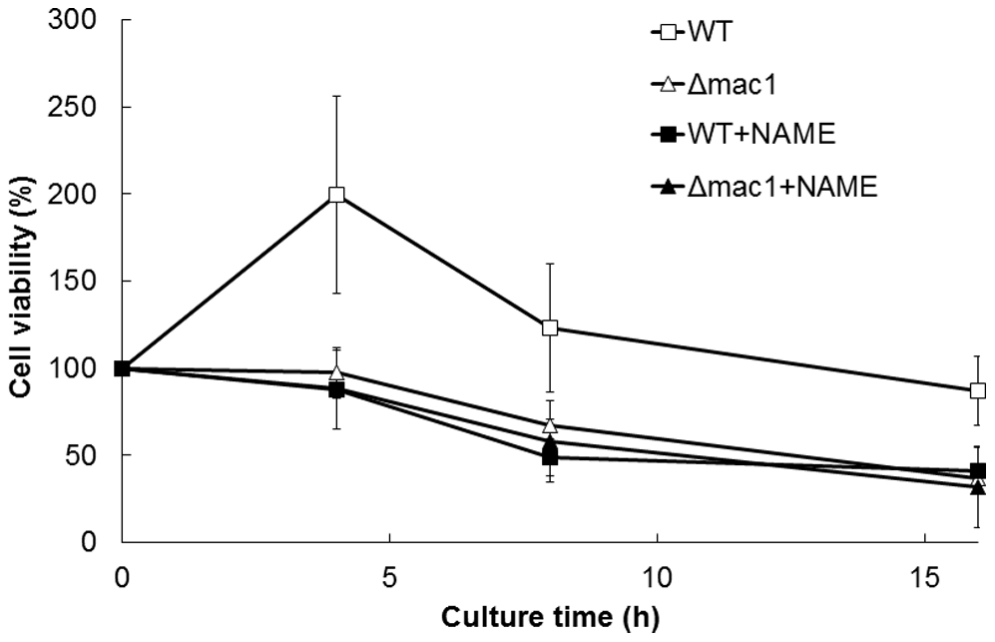
Cell viability under high-temperature stress conditions. Viability of *S. cerevisiae* L5685 or L5685*Δmac1* cells was measured after exposure to high temperature in the presence (100 mM) or absence of NAME. Cell viability was expressed as percentages, calculated as follows: (no. of colonies after exposure to high temperature stress (39°C))/(no. of colonies after exposure to non-stress condition (25°C))×100. The values are the means and standard deviations of at least two independent experiments.

We further examined the effect of *MAC1* and NO on the survival rate of yeast after exposure to high temperature (Fig. 4). The cell viability of *mac1*-disrupted cells after exposure to 39°C was significantly lower than that of wild-type cells. Interestingly, when NAME was added, wild-type cells were more sensitive to high temperature than were the untreated cells. In contrast, NAME treatment did not affect the survival rate of *mac1*-disruptant cells. These results indicate that NO contributes to high-temperature stress tolerance in a manner dependent on Mac1.

### NO produced under high-temperature stress conditions increases the intracellular copper content in a Mac1-dependent manner

**Figure 5:**
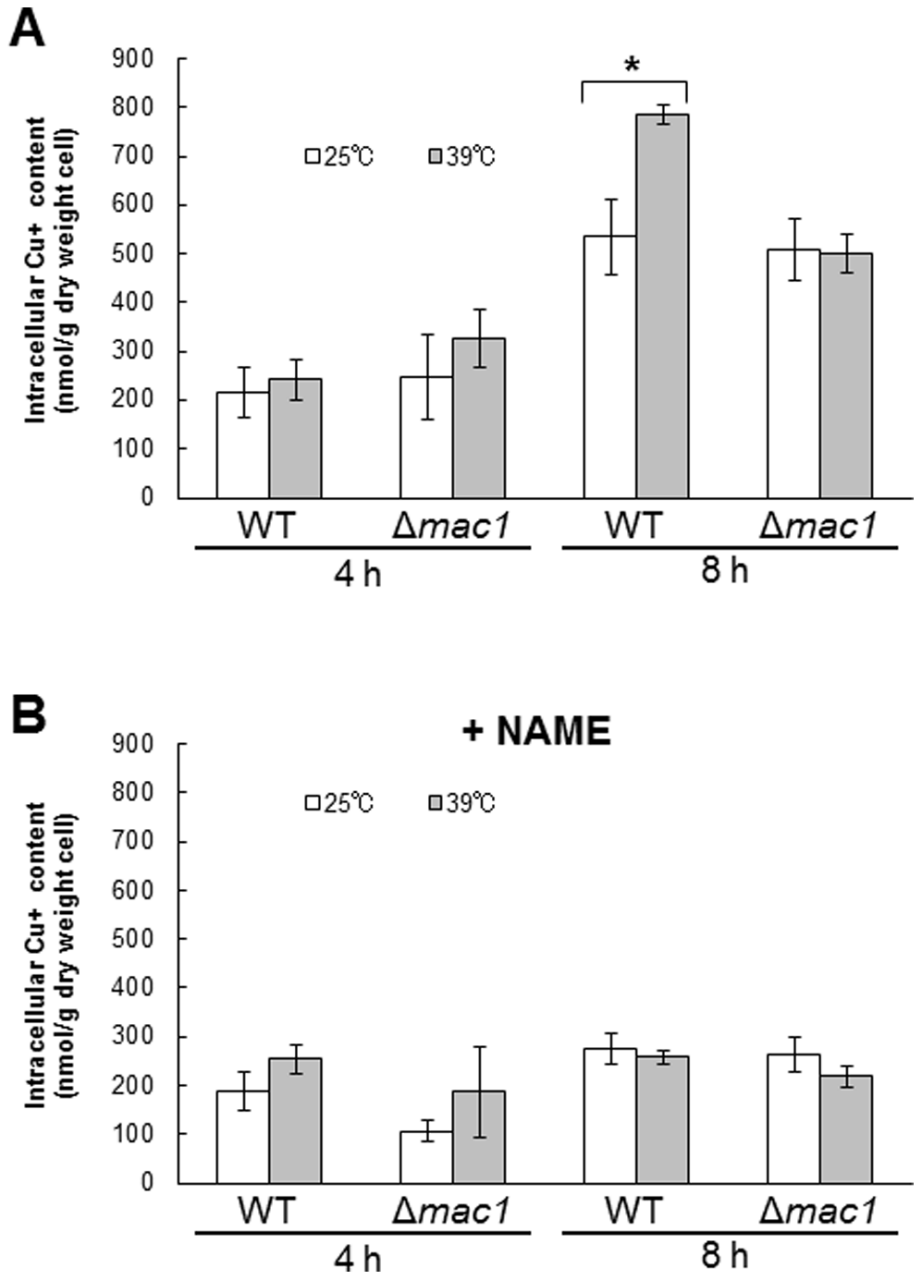
Intracellular copper content. The intracellular copper content in *S. cerevisiae* L5685 or L5685*Δmac1* cells was measured before or after exposure to 39°C in the absence (A) or presence (B) of 100 mM NAME. The values are the means and standard deviations of three independent experiments. *p<0.05 by Student's *t* test.

The overexpression of *CTR1* has been reported to induce the accumulation of copper ions in yeast cells [25]. We determined the intracellular copper content after exposure to high temperature (Fig. 5). When wild-type cells were exposed to 39°C for 8 h, copper level was increased by approximately 50% as compared with that of control cells (25°C). However, such an increase was not observed in *mac1*-disrupted cells (Fig. 5A). On the other hand, copper ions were not accumulated in response to high temperature in either wild-type or *mac1*-disrupted cells when cells were treated with NAME (Fig. 5B). These results indicate that NO-mediated Mac1 activation induces copper accumulation in yeast cells under high-temperature stress conditions.

### NO produced under high-temperature stress conditions enhances Sod1 activity through the activation of Mac1

**Figure 6:**
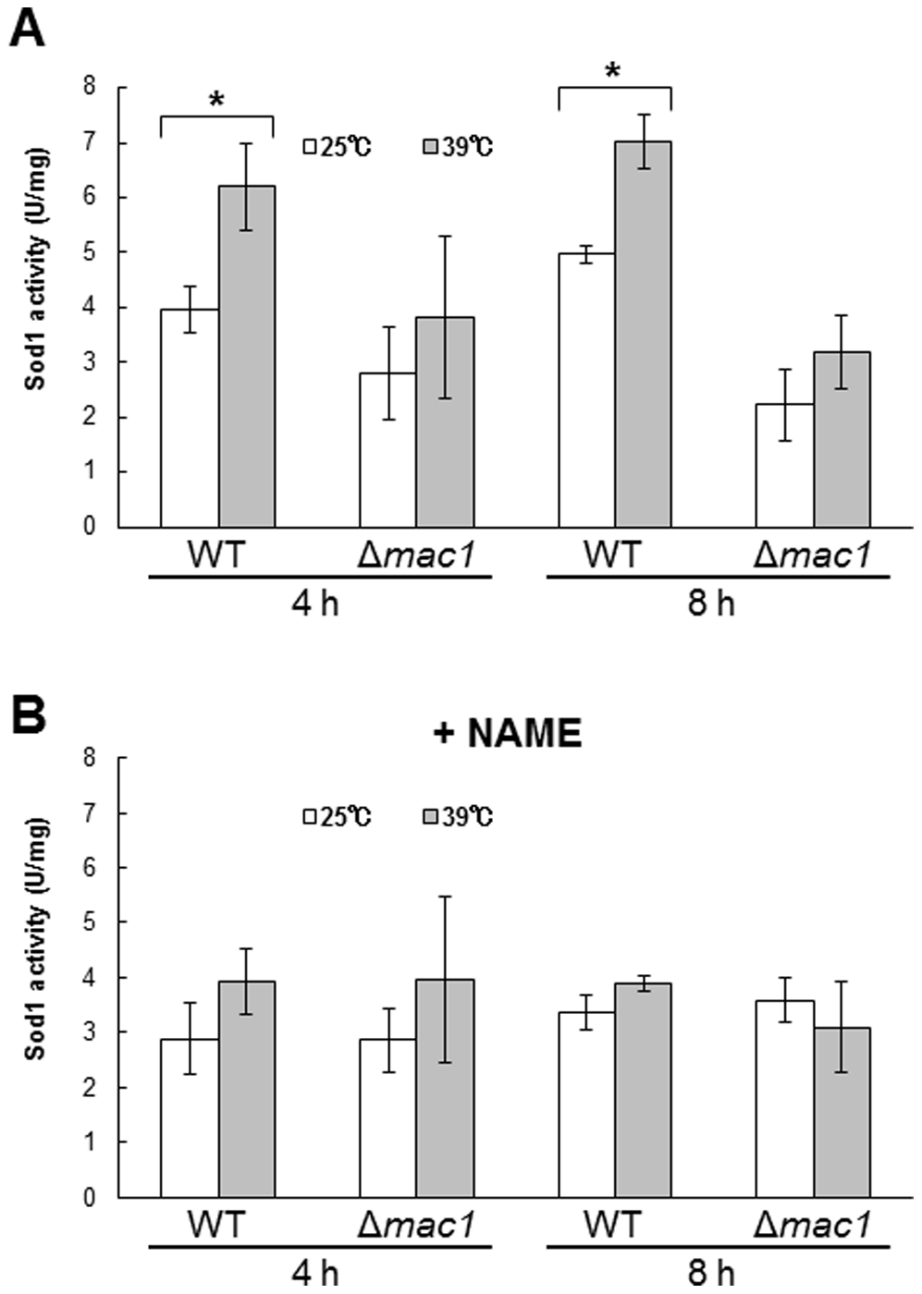
The activity of Cu,Zn-superoxide disumutase Sod1. Sod1 activity in *S. cerevisiae* L5685 or L5685*Δmac1* cells was measured before or after exposure to 39°C in the absence (A) or presence (B) of 100 mM NAME. The values are the means and standard deviations of three independent experiments. *p<0.05 by Student's *t* test.

Generally, living organisms have defense systems against superoxide generation. In *S. cerevisiae* cells, there are two different enzymes that catalyze the dismutase reaction of superoxide, Sod1 and Sod2. The cytosolic Sod1 requires copper and zinc ions as cofactors, whereas Sod2, which localizes in the mitochondria, requires manganese as a cofactor [35]. It was reported that Cu(I) insertion into Sod1 is indispensable for its enzymatic activity [36]. The disruption of *SOD1* has been shown to confer thremosensitivity to yeast cells [37], suggesting that the reduction of superoxide anion generated under heat-shock conditions is important for stress tolerance. Therefore, we measured the activity of Sod1 (Fig. 6). An approximately 50% increase in Sod1 activity was also observed in wild-type cells after incubation at 39°C for 4 h and 8 h. In contrast, Sod1 activity was not significantly elevated in *mac1*-disrupted cells (Fig. 6A). NAME treatment also inhibited the increase in Sod1 activity at 39°C (Fig. 6B). It should be noted that the transcription level of *SOD1* after exposure to stress was not decreased by the disruption of *MAC1* or NAME treatment (Fig. 3). The exogenous NO treatment also enhanced the activity of Sod1 without any increase in the protein level of Sod1 (Fig. S2). These results suggest that NO-dependent Mac1 activation increases Sod1 activity by providing Cu(I) to the Sod1 protein.

### NO-mediated antioxidative mechanism through the activation of Mac1

**Figure 7:**
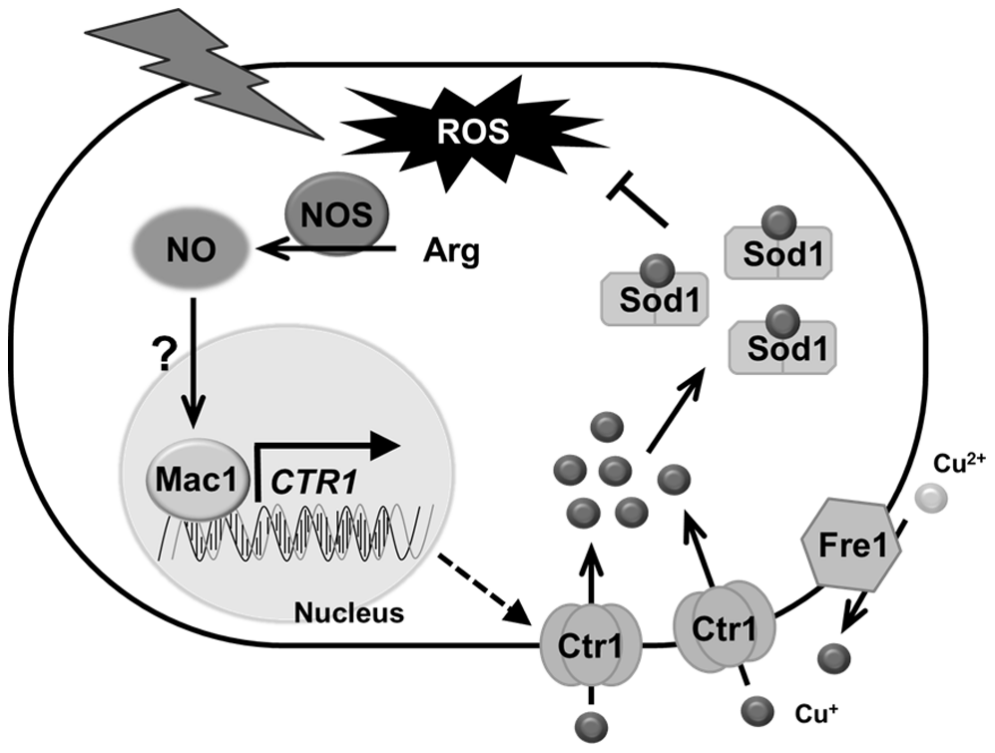
A proposed model for the antioxidative mechanism through Mac1 activated by NO. When yeast cells are exposed to high-temperature stress, NO is produced from arginine by NOS-like activity. NO-mediated Mac1 activation through its post-translational modification upregulates the expression of *CTR1*, which encodes a copper transporter. Protein name: Fre1, cupric reductase; Ctr1, copper ion transporter; Sod1, Cu,Zn-superoxide dismutase; Ccs1, copper chaperone; Mac1, transcription factor responsible for copper metabolism.

From the results shown here, we propose a novel antioxidative mechanism through the activation of Mac1 by NO (Fig. 7). Temperature up-shift induces the generation of ROS including superoxide anion and also NO production from arginine requiring Tah18 [8]. NO activates Mac1 via post-translational modification such as *S*-nitrosylation and/or phosphorylation. The active form of Mac1 binds to the promoter region of *CTR1* to induce its expression. Cu(I) provided from the reduction of Cu(II) catalyzed by cupric reductase is imported into cells due to the increase in the protein level of Ctr1. Sod1, to which the cytosolic copper chaperone Ccs1 delivers Cu(I), is then converted from the apo-form to the holo-form to express its full activity. Finally, the enhanced Sod1 activity degrades superoxide anion by its dismutase reaction, conferring stress tolerance to yeast cells.

## Discussion

Yeast cells have numerous defense mechanisms against oxidative stress, such as antioxidative enzymes, ROS scavengers, and transcription factors. Recently, we discovered a novel antioxidative mechanism mediated by NO in *S. cerevisiae* [8]. When yeast cells are exposed to any environmental stresses that facilitate the generation of ROS in the cell, the intracellular arginine level is increased and then arginine can be converted into NO and citrulline by NOS-like activity dependent on the Tah18 protein [8]. Although NO confers oxidative stress tolerance to yeast cells, the mechanism underlying NO-mediated stress tolerance has been unclear. In this study, we revealed a novel mechanism of antioxidative stress resistance mediated by NO, which is one of the downstream pathways of NO. This study is the first to report the molecular mechanism for a novel antioxidative stress resistance mediated by NO in yeast cells.

*S. cerevisiae* has the *YHB1* gene encoding flavohemoglobin that detoxifies excel level of NO [38]. However, our data suggest that NO functions as a signaling molecule for antioxidative mechanism under high-temperature stress conditions. NO was shown to contribute to cell protection against high-temperature stress eventually by increasing Sod1 activity. A previous study showed the disruption of *SOD1* conferred thermosensivity to yeast cells [37], suggesting that high-temperature stress induces the generation of superoxide anion that damages cells. Therefore, we think that increased NO leads to activation of Sod1, which directly reduces the level of superoxide anion (Fig. 7).

The exogenous NO treatment upregulated the transcription of many genes involved in copper uptake and its utilization, including *CTR1*, *FRE1*, and *FRE7* (Fig. 1). These genes are under the control of the transcription factor Mac1. Interestingly, some of Mac1-regulated genes are not induced in response to NO. Although the reason is currently unclear, this suggests that the manners of activation and/or target specificity of Mac1 under copper starvation conditions are different from those induced by NO. Furthermore, among Mac1-regulated genes, *CTR1* is the only gene that is strongly induced under high-temperature stress conditions in a Mac1- and NO-dependent manner. The induction of *FRE1* is dependent on Mac1 but not on NO (Fig. 3). Judging from the previous report on intracellular NO generated in response to H_2_O_2_, NO concentration under high-temperature stress conditions was suggested to be lower than that of SNAP used in this study (100 µM) [20]. Another copper-related transcriptional factor, Ace1, also binds copper ions with its cysteine residues in a manner similar to Mac1 [31] and is required for tolerance against copper toxicity. Interestingly, NO regulates Ace1 in a dose-dependent manner [39]. These results also suggest that the effect of NO on Mac1 is strictly dependent on its concentrations. On the other hand, it is possible that Mac1 is oxidized by ROS under high-temperature stress conditions because it has many cysteine residues. Thus, intracellular NO level and oxidative state might control the activation mechanism and/or target specificity of Mac1.

We showed that the cell viability, the expression level of *CTR1*, the intracellular copper content, and the activity of Sod1 were increased in a Mac1- and NO-dependent manner under high-temperature stress conditions. These results led us to propose a novel antioxidative mechanism mediated by NO and Mac1 (Fig. 7). The expression level of *CTR1* (Fig. 3) and the activity of Sod1 (Fig. 6) were shown to increase after exposure to 39°C for 4 h. The effect of NO and Mac1 on the cell viability was also observed after temperature up-shift for 4 h (Fig. 4). However, the NO-dependent increase in intracellular copper content was observed after cultivation at 39°C for 8 h (Fig. 5). These results suggest that the induction of *CTR1* by the active form of Mac1 occurs after exposure to high temperature for 4 h, and then copper enters cells through Ctr1. Subsequently, Sod1 activity is enhanced and yeast cells finally acquire a defense mechanism for high-temperature stress after 8 h. However, a significant increase in Sod1 activity and the effect of NO on the cell viability were already observed without any copper accumulation after 4 h. This may be due to the additional stress-tolerance mechanism requiring NO-mediated Mac1 activation, which is independent of copper accumulation. It has been reported that incorporation of Cu(I) into the apoenzyme of Sod1 requires the copper chaperone Ccs1. In silico analysis to predict the transcription factor for *CCS1* using YEASTRACT (http://www.yeastract.com) [40]–[43] revealed that Mac1 is a potential transcription factor for *CCS1*. This result raises the possibility that Mac1 activated by NO induces the expression of *CCS1* and Ccs1 catalyzes the incorporation of Cu(I), which is accumulated at the basal level, into the apoenzyme of Sod1 after exposure to stress for 4 h independently of copper uptake from environment.

It was observed that the intracellular copper content increased even under non-stress conditions (25°C) and this increase was clearly canceled by NAME treatment (Fig. 5), suggesting that NO is involved in copper accumulation under normal conditions with cultivation. The disruption of *MAC1* did not change copper content, indicating that copper accumulation under normal conditions is independent of Mac1. In addition to Mac1, NO may regulate some copper transporters, cupric reductases, and transcription factors responsible for copper metabolism under non-stress conditions.

Here, we demonstrated that NO activated Mac1. NO did not affect the expression level of *MAC1* (Fig. 3), thus Mac1 is likely to be activated through post-translational modification such as *S*-nitrosylation or phosphorylation. NO binds to and activates sGC, leading to an increase in cGMP level in mammalian cells. However, genome-wide analysis failed to identify sequences that are homologous to mammalian sGC in *S. cerevisiae*. One can raise the possibility that NO directly activates Mac1 through *S*-nitrosylation. The past study indicated that Mac1 is activated by releasing copper ions under copper starvation conditions, whereas the inactive form of Mac1 binds eight copper ions by its cysteine residues [44]. *S*-Nitrosylation of cysteine residues responsible for copper binding should decrease their ability to coordinate copper, suggesting that NO activates Mac1 through *S*-nitrosylation of its cysteine residues. On the other hand, it is known that yeast has sGC activity, which is increased by NO treatment [45], despite the absence of its orthologue. Yeast is suggested to have a protein possessing the same function as sGC without its sequence similarity and to have a signaling pathway including NO/sGC/cGMP system. cGMP acts as a second messenger activating cGMP-dependent protein kinases, leading to the subsequent protein phosphorylation cascade [46]. It was also shown that the activation of Mac1 requires its phosphorylation [30]. These facts raise another possibility with respect to the activation of Mac1. NO could enhance sGC activity to increase the cGMP level, and then the activated protein kinases by cGMP could trigger Mac1 activation directly through its phosphorylation or indirectly through protein kinase cascade. More biochemical analyses will be needed to clarify the mechanism.

Although Sod1 was reported to be necessary for the activation of Mac1 under the copper-depleted conditions [29], we here presented evidence that Mac1 induces the activation of Sod1 in response to high temperature. It is possible that, by causing Mac1 and Sod1 to mutually activate each other, the Mac1-Sod1 pathway has a positive feedback system to enhance the NO-triggered signal for oxidative stress tolerance.

NO exerts a dual role on the cell, including cell death when present at high concentrations and cell protection when present at low concentrations [47]. Here we revealed an NO-mediated cell protection system through the activation of Mac1. NO may also exhibit effects on the Mac1 activity opposite those of Ace1, because Ace1 is regulated through the binding of copper to its cysteine residues in response to high copper concentrations in a manner similar to Mac1 [31]. If an excess level of NO inactivates Mac1 as speculated above, such a situation should confer stress sensitivity, leading to cell death. NO-mediated stress tolerance through the regulation of Mac1 might be one of the mechanisms to explain the dual effect of NO.

## Supporting Information

Figure S1 **NO production in yeast cells.** NO production was shown as fluorescence from DAF-FM DA in *S. cerevisiae* L5685 or L5685*Δmac1* cells treated or untreated with high temperature (39°C) in the presence or absence of NAME. All pictures were taken in the same exposure time.
(DOC)

Figure S2 **Sod1 activity of yeast cells treated with the exogenous NO.** (A) Sod1 activity was measured in *S. cerevisiae* BY4741 or BY4741*Δsod1* cells treated or untreated with SNAP. The values are the means and standard deviations of three independent experiments. *p<0.001 by Student's *t* test. (B) Total proteins (1 µg) in the soluble extract were subjected to 12.5% SDS-polyacrylamide gel electorphoresis, and Sod1 and the internal control protein glyceraldehyde-3-phosphate dehydrogenase (GAPDH) were detected using each antibody.
(DOC)

Table S1 **Primers used in this study.**
(DOC)
