# Supplementary material for: Nitric Oxide-Mediated Antioxidative Mechanism in Yeast through the Activation of the Transcription Factor Mac1
Source: PLoS One. 2014 Nov 25;9(11):e113788. doi: 10.1371/journal.pone.0113788 (PMC4244153; doi:10.1371/journal.pone.0113788)
Supplement: Figure S1 — NO production in yeast cells. NO production was shown as fluorescence from DAF-FM DA in S. cerevisiae L5685 or L5685Δmac1 cells treated or untreated with high temperature (39°C) in the presence or absence of NAME. All pictures were taken in the same exposure time. (DOC) [file pone.0113788.s001.doc]

**Figure S1. NO production in yeast cells.**
