# Supplementary material for: Nitric Oxide-Mediated Antioxidative Mechanism in Yeast through the Activation of the Transcription Factor Mac1
Source: PLoS One. 2014 Nov 25;9(11):e113788. doi: 10.1371/journal.pone.0113788 (PMC4244153; doi:10.1371/journal.pone.0113788)
Supplement: Figure S2 — Sod1 activity of yeast cells treated with the exogenous NO. (A) Sod1 activity was measured in S. cerevisiae BY4741 or BY4741Δsod1 cells treated or untreated with SNAP. The values are the means and standard deviations of three independent experiments. *p<0.001 by Student's t test. (B) Total proteins (1 µg) in the soluble extract were subjected to 12.5% SDS-polyacrylamide gel electorphoresis, and Sod1 and the internal control protein glyceraldehyde-3-phosphate dehydrogenase (GAPDH) were detected using each antibody. (DOC) [file pone.0113788.s002.doc]

**Figure S2. Sod1 activity of yeast cells treated with the exogenous NO.**
