# Supplementary material for: Nitric Oxide-Mediated Antioxidative Mechanism in Yeast through the Activation of the Transcription Factor Mac1
Source: PLoS One. 2014 Nov 25;9(11):e113788. doi: 10.1371/journal.pone.0113788 (PMC4244153; doi:10.1371/journal.pone.0113788)
Supplement: Table S1 — Primers used in this study. (DOC) [file pone.0113788.s003.doc]

**Table S1.** Primers used in this study

| Name | Sequence (5’-3’) | Description |
| --- | --- | --- |
| CTR1-RT01-F | GGTAACTGCCAATGTGGTAGACAT | RT qPCR, ChIP analysis |
| CTR1-RT01-R | ATCGGCAACAGCAATTGGAT | RT qPCR, ChIP analysis |
| CTR3-RT01-F | CATTGCCAGATCTTGGAGAAATG | RT qPCR |
| CTR3-RT01-R | ACAGCCGATGCAAGAACCA | RT qPCR |
| FRE1-RT01-F | CCACATATCGCTAAGCTAAAAAGAAA | RT qPCR, ChIP analysis |
| FRE1-RT01-R | GCCGCAACACCCAAACC | RT qPCR, ChIP analysis |
| FRE7-RT01-F | TGCTGACATCCACTCCGAACT | RT qPCR |
| FRE7-RT01-R | GTGTCGCTGCCTGCGATT | RT qPCR |
| IRC7-RT01-F | GGCTCGGAAATCGAGATGAG | RT qPCR |
| IRC7-RT01-R | TCCGGGACAATCTTCAAAGG | RT qPCR |
| REE1-RT01-F | CGGAAATGCTCGCGATTT | RT qPCR |
| REE1-RT01-R | GAATCCTCAAAACGCCCTTTT | RT qPCR |
| YHB1-RT01-F | TCAAAAGGTTGGCGCACAA | RT qPCR |
| YHB1-RT01-R | CTTAGCCGCTGCCAAAACA | RT qPCR |
